# All-optical majority gate based on an injection-locked laser

**DOI:** 10.1038/s41598-019-51025-y

**Published:** 2019-10-10

**Authors:** Tuomo von Lerber, Matti Lassas, Vladimir S. Lyubopytov, Lauri Ylinen, Arkadi Chipouline, Klaus Hofmann, Franko Küppers

**Affiliations:** 1 Photonics Lab, Technische Universität Darmstadt, Darmstadt, Germany; 2 Department of Mathematics and Statistics, University of Helsinki, Helsinki, Finland; 3 Telecommunication Systems Dept., Ufa State Aviation Technical University, Ufa, Russian Federation; 4 Integrated Electronic Systems Lab, Technische Universität Darmstadt, Darmstadt, Germany; 5 Center for Photonics and Quantum Materials, Skolkovo Institute of Science and Technology, Moscow, Russian Federation

**Keywords:** Computer science, Lasers, LEDs and light sources, Photonic devices

## Abstract

An all-optical computer has remained an elusive concept. To construct a practical computing primitive equivalent to an electronic Boolean logic, one should utilize nonlinearity that overcomes weaknesses that plague many optical processing schemes. An advantageous nonlinearity provides a complete set of logic operations and allows cascaded operations without changes in wavelength or in signal encoding format. Here we demonstrate an all-optical majority gate based on a vertical-cavity surface-emitting laser (VCSEL). Using emulated signal coupling, the arrangement provides Bit Error Ratio (BER) of 10^−6^ at the rate of 1 GHz without changes in the wavelength or in the signal encoding format. Cascaded operation of the injection-locked laser majority gate is simulated on a full adder and a 3-bit ripple-carry adder circuits. Finally, utilizing the spin-flip model semiconductor laser rate equations, we prove that injection-locked lasers may perform normalization operations in the steady-state with an arbitrary linear state of polarization.

## Introduction

During the past four decades, successful scaling of CMOS technology has enabled a multitude of computing devices, such as mobile phones and personal computers, which contain transistors numbering from hundreds of millions to billions. Recently, however, miniaturization of the field-effect transistors has become increasingly difficult because of the quantum mechanical effects, the challenges of doping, and the limits of thermal dissipation. Consequently, the computing community has been searching for alternative processing paradigms, such as spintronic majority-gate circuits^1^.

Optical computing has been studied as an alternative to silicon-based technologies. Optical processors do exist, but they have found only a few applications outside of Fourier transforms, pattern recognition, and optical correlation^2^; yet, optical processing remains an active field of research still today^3^. The fundamental challenge in optical computing is the nature of photons that do not interact with each other in the vacuum but require a nonlinear medium. Optical transistors have been proposed in a number of arrangements, such as in atomic ensembles^4–6^ and in a semiconductor microcavity^7^. Optical gates and switches have been studied with various nonlinear components, such as semiconductor optical amplifiers^8–12^, and waveguides and resonators^13–17^. Further on, metamaterials have been explored for both analogue and digital computations^18–20^.

A wide variety of injection-locked laser based signal processing arrangements have been proposed for telecommunications and all-optical computing. For instance, in telecommunications, the lasers have been used for enhancement of modulation bandwidth^21^, carrier phase recovery and regeneration^22^, phase-preserving amplitude limiting^23^, and orthogonal separation of amplitude and phase^24^. In the field of all-optical computing, the injection-locked gain-modulated lasers (later referred as the *gain-modulation method*) have been used to demonstrate a number of logic gates (NOT^25–27^, NOR^25–28^, AND^26,28^, OR^26,28^, NAND^26,29–31^, XNOR^29^, XOR^28^), switches^32–36^, a bit-error monitor^37^, a digital adder^29^, a comparator^38^, flip-flops^39–41^, and data packet header processing^33,34^.

Arrangements of the gain-modulation method share the same fundamental principles of operation. Namely, the injected data signal takes part in mode-competition process inside the laser active medium, where the emission is altered by the interaction between the electromagnetic fields of the data signal (of some wavelength *λ*_1_), naturally occurring intracavity fields, and other possibly injected fields (control signals, other data streams etc), each possessing its own particular wavelength *λ*_*i*_. This mode-competition process is strongly nonlinear and provides useful phenomena, such as hysteresis that can be used for switching. The changes in the mode-competition process, and thus changes of gain for a specific wavelength of light, rely on mutual variations of the injected powers. That is, the injected light, the data and the control signals alike, are amplitude-shift keyed (ASK). When the laser emission is coupled with a suitable bandpass filter, the output will manifest ASK encoded power levels, yet, often with a converted wavelength. Given proper management of the wavelengths and injected signal powers, operations can be combined and even complex functionalities can be realized.

When aiming for an integrated large scale signal processing, a particular challenge of many optical processing schemes is to cascade operations economically. A practical optical cascade requires logic level restoration, unaltered wavelength of operation, and unmodified encoding of signal^42,43^.

In this Paper we propose an all-optical computing primitive based on normalization operations of injection-locked lasers. Based on universal approximation theorem the normalization operations can be used to construct an arbitrary continuous function $f:{{\mathbb{R}}}^{n}\to {{\mathbb{R}}}^{m}$, with any given accuracy and we demonstrate a Boolean logic with a VCSEL-based all-optical majority gate at a rate of 1 GHz with BER of 10^−6^ without changes in the operating wavelength or in the signal encoding format, as required for practical cascadability. Using spin-flip model laser rate equations, we show that in steady-state a weakly injection-locked laser may perform a two-dimensional complex normalization operation of linearly polarized electric fields (Supplementary S1) and we simulate an optical full adder and a 3-bit ripple-carry adder (Supplementary S2).

## Operating Principle

An injection-locked slave laser is known to adopt and retain the phase of the master, while it will quench variations in injected signal amplitude. Qualitatively, this can be shown with a pair of rate-equations^44^

1 $${\gamma }_{c}^{-1}\dot{E}=-\mathrm{(1}+i\alpha )(1-N)E+{u}_{\eta }$$

2 $${\gamma }^{-1}\dot{N}=-\mathrm{(1}+|E{|}^{2})N+\mu ,$$

where *E* and *u*_*η*_ are the slowly varying complex amplitudes of the emitted and injected fields, respectively; *N* is the normalized total carrier density, *γ*_*c*_ is the cavity photon decay rate, *γ* is the carrier recombination rate, *α* is the linewidth enhancement factor, and *μ* is the normalized pumping rate. Here we assume a linearly polarized emission out of, and the same state of polarization injection into a laser with absence of polarization anisotropies. An increase in amplitude of the emitted field *E* of Eq. (1) leads to a decrease in carrier density *N* of Eq. (2), which in turn will couple negatively back to the field *E*. Thus, under constant pumping above the lasing threshold (*μ* > 1) the system will approach steady-state ($\dot{E}=0$ and $\dot{N}=0$) where the perturbations of the optical injection *u*_*η*_ are diminished at the emitted output. A more rigorous steady-state analysis of spin-flip model rate equations are presented in Supplementary equations S1.

**Figure 1:**
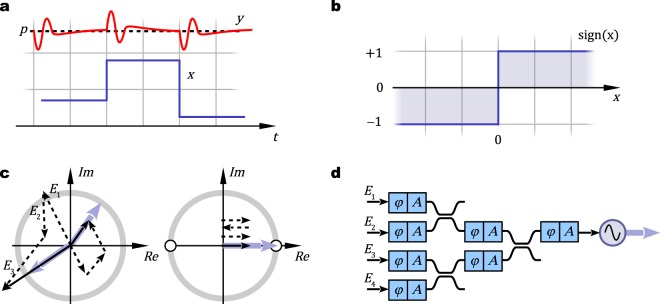
Principle of operation. (**a**) Slave oscillator amplitude |*y*| (red line) approaches a constant *p* at steady-state despite the fluctuations in the master |*x*| (blue). (**b**) Normalization operation is akin to signum-function for a real-valued input. (**c**) On complex plane, summations of multiple input signals (dashed arrows) are normalized (blue arrows) on unit circles. An odd number of combined binary signals have possible outputs shown with small white circles at {−1, +1}. (**d**) An illustration of an integrated optical circuit that combines multiple inputs that are injected into a laser. Optical paths are equipped with individually adjustable phase-shifts *φ* and losses *A*.

In general, design of sophisticated circuits requires an operating principle that can be described with a simple and succinct formulation. For example, digital electronic circuitry is regularly expressed with Boolean algebra that allows elaborate manipulations and use of ready-made libraries in design of complex circuitry. To that end we purposefully express the above described approximation of injection-locked lasers with a normalization operation as

3 $$y=p\frac{x}{\Vert x\Vert },$$

where *x* and *y* are complex amplitudes of the master and slave electric fields, respectively; and *p* is a dimensionless amplification factor determined by the slave. Figure 1a schematically illustrates amplitudes of a master (blue line) and a weakly-coupled slave (red line). After a perturbation, the amplitude of the slave returns to a constant while the phase remains locked with the master. The normalization operation can be further rendered into the signum function (see Fig. 1b) if the phase of the master equals to an integer of *π*.

Our treatment focuses on optical systems with a single linear state of polarization, yet we readily acknowledge the possibility for two orthogonal states of polarizations in which case the signals would be expressed with two-dimensional complex amplitude vectors and normalizations of the same (see Supplementary Equation S1).

Normalization of a phase encoded signal is akin to phase preserving limited amplification that has been proposed for regeneration in telecommunications systems^22^. As the name implies, the purpose of regeneration is to remove phase and amplitude noise from a channel while not changing the actual information content. In contrast, in computations the input is coming from more than a single source. Figure 1c depicts two phasor diagrams with superpositions of multiple signals and their normalizations. When the input contains an odd number of binary signals, their normalized superposition will always result in a binary output of −1 or +1. An even number of phase encoded binary inputs could potentially be problematic, because the amplitude of their superposition could be zero, which would result in a non-lock and unstable operation of the laser.

To combine signals from various sources, a possible integrated optics realization may contain a number of directional couplers that connect into a laser (see Fig. 1d). A collection of such circuit primitives can be used to construct a desired operations as discussed below. In following it is assumed that the master and the slave lasers have the same angular frequencies, the signal phases stay controlled, and the oscillators in a cascaded system have nonreciprocal flow of information, i.e., a master controls slaves but not the opposite.

From a practical viewpoint, when the principles of operation of the normalization method and the gain-modulation method are compared, we may observe differences in (i) wavelength management, (ii) control of signal phases, and (iii) signal encoding formats.

(i) **Single wavelength vs. multiwavelength**. Using the normalization method, the coupled inputs injected into a laser require the same wavelength, while the gain-modulated logic gate requires a multiwavelength input (except for the NOT gate). Thus, a system using the normalization method is assumed to have a common reference with sufficiently high coherence, while the gain-modulation method requires multiple light sources.
(ii) **Controlled phases vs. relaxed phase control**. The normalization method requires mutually controlled phases of input signals, while the gain-modulation method is indifferent to phases of lightwaves.
(iii) **Signal encoding in full complex plane vs. positive real values, usually Boolean**. The normalization operation is agnostic to signal encoding format, given that the input amplitude variation is within the dynamic range of the laser. Thus, the full complex plane can be used for the signal encoding. The gain-modulation method is indifferent to signal phases and, therefore, the signal encoding is inherently limited to positive real values, usually Boolean.

## All-Optical Majority Logic

**Figure 2:**
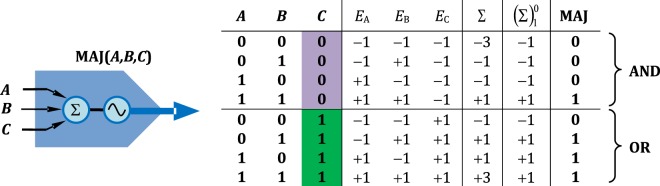
Truth table of the majority gate. The table contains symbol values (*A*, *B*, *C*), accompanied complex electric field amplitudes (*E*_*A*_, *E*_*B*_, *E*_*C*_), the sum of the electric field amplitudes (∑), and the normalized output ${(\Sigma )}_{1}^{0}$; the normalization operation is denoted as ${(x)}_{p}^{0}\equiv px/\Vert x\Vert$.

We demonstrate the normalization operation with a majority logic^45–48^ using an injection-locked laser and an emulated input of three optical signals. The input signals *A*, *B*, and *C* are phase encoded such that the binary numbers of 1 and 0 relate to phase-shifts of 0 and *π*, and complex electric field amplitudes of +1 and −1, respectively. As known in the art, when one of the inputs, now the signal *C*, is set to 0 (hence the phase shift is *π* and the related electric field amplitude is −1), the majority function will produce the Boolean AND(*A*, *B*); and *C* = 1 will produce the OR(*A*, *B*) operation as shown in Fig. 2.

We experimented the majority gate using a Vertical-Cavity Surface-Emitting Laser (VCSEL), which was injected with an emulated superposition of phase encoded signals of *A*, *B*, and *C*; where the signals *A* and *B* were uncorrelated and randomly generated, and the signal *C* was switched between 1 and 0 for each successive operation. The hybrid encoded signal *A* + *B* + *C* had relative intensity variation of 1:9; that is, having the extinction ratio (ER) of 9.54 dB, and phases of 0 and *π*. The VCSEL output was connected to a Bit Error Ratio Tester (BERT) that counted the errors. For detailed description of the measurement setup, see the Methods section.

**Figure 3:**
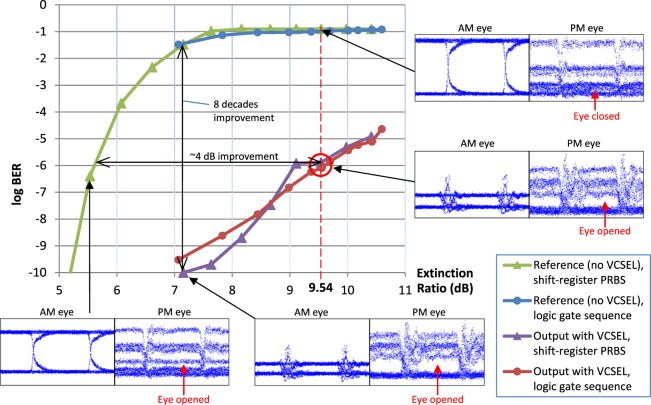
Measured BER with and without slave VCSEL for varying ER. Red circle: the BER at the operation point of the majority logic gate. The increase of the parasitic ER increases the BER, yet, the inclusion of the injection-locked VCSEL decreases the BER up to 8 decades, or 4 dB in terms of the ER. Eye diagrams of the AM and PM signals are shown for some points of interest. The quenching of the unwanted variation in amplitude is evident in presence of the VCSEL. The eye diagram of the reference signal at ER of 5.5 dB shows that the emulated hybrid encoded signal had good quality at current data rate.

**Figure 4:**
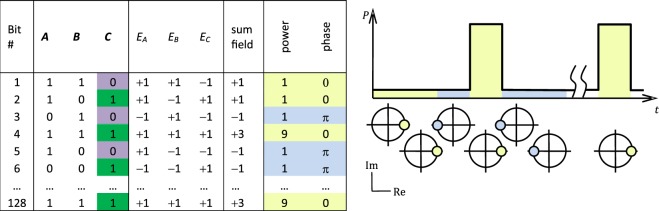
Symbols and electric fields of the emulated three signal sum input.

The slave VCSEL’s ability to quench the amplitude modulation was measured first by gradually increasing the ER of the hybrid amplitude-phase encoded signal. The BER was measured with and without the presence of the slave VCSEL (see Fig. 3). In absence of the VCSEL the BER is >10^−3^ when ER is at 6.5 dB, and when ER is above 7.5 dB, the BER saturates. As evident, an uncompensated receiver performance is severely compromised with increase of the amplitude modulation. When the slave VCSEL is present, the BER is improved by 8 decades at ER = 7.0 dB; or at fixed BER of 10^−6^ the improvement in ER sustainability is 4 dB. All BER measurements were performed under constant receiving power. The BER improvement, effectively a decrease of information entropy, is a proof of a successful injection-locking of the VCSEL, both in terms of the amplitude and the phase. The BER readings are similar when the hybrid amplitude-phase encoding was changed to logic gate sequence feed of Fig. 4. As noted above, the superposition of the inputs *A*, *B*, and *C* had the ER of 9.54 dB, which in the current setup is translated into BER of 10^−6^.

To build a practical optical computing circuit, one has to ensure cascadability of the scheme^42^. We investigate cascadability of the majority gates first with a simulation of a full adder, and further on, a simulation of a 3-bit ripple-carry adder based on aforementioned full adders. The schematic integrated optical circuits, simulation models, and results are presented in Supplementary Information.

## Discussion

We have proposed an all-optical computing scheme based on normalization operations of injection-locked lasers and demonstrated the scheme with the optical majority logic, where the input and output signals share the same wavelength, the same optical power, and the same signal encoding format; all prerequisites for elaborate circuit designs. Semiconductor lasers are attractive candidates for all-optical processing because they offer scalable wafer level fabrication and integration, which enables inter- and intra-chip optical communications that bypasses expensive electro-optic conversions.

## Methods

**Figure 5:**
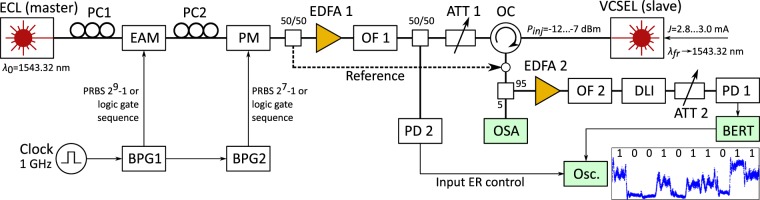
Schematic illustration of the measurement setup. Abbreviations: ECL – external cavity laser; PC – polarization controller; EAM – electro-absorption modulator; PM – phase modulator; BPG – bit pattern generator; EDFA – Erbium doped fibre amplifier; OF – optical filter; ATT – variable optical attenuator; OC – optical circulator; PD – p-i-n photodiode; OSA – optical spectrum analyser; DLI – delay line interferometer; BERT – bit error rate tester; Osc. – digitizing oscilloscope.

We emulated the combined input of *A* + *B* + *C* with a tandem of an external cavity laser (ECL) with a linewidth of 150 kHz (OSICS ECL 1560/P6), an electro-absorption modulator (EAM), and a phase modulator (PM) that together produced the desired multilevel hybrid encoding (see Figs 4 and 5). Similar emulation of multiple signals is common in telecommunications, particularly in orthogonal frequency-division multiplexing (OFDM) systems.

To experiment the logic gate regime, the bit streams of *A* and *B* were each given 128 randomly generated bits (not using linear feedback shift registers, but a computer-generated sequence of Fig. 4) and the signal *C* was sequentially switched between 0 and 1 for each operation. The respective multilevel phase-modulated signal had relative power levels of 1 and 9, i.e., the extinction ratio (ER) of 9.54 dB, and the phase shifts of 0 and *π*. The EAM and PM were each controlled by separate bit pattern generators (BPG) Anritsu MP1755A and Advantest D3186, respectively, that provided independent bit patterns at a rate of 1 Gbit/s. The BPGs were synchronized in time such that the modulated optical amplitude and phase symbols coincided. The PM output was coupled to an erbium-doped fibre amplifier (EDFA), an optical tunable bandpass filter (OF), and a variable attenuator (ATT) in order to control and optimize the optical power of the seeding signal. During the preparation, the setup was experimented with shift-register-based PRBSs with dissimilar word lengths of 2^9^ − 1 and 2^7^ − 1 that were fed to EAM and PM, respectively.

The seeding signal was coupled into the slave laser (commercial single-mode VCSEL by VERTILAS GmbH) via an optical circulator (OC), which directed the returning signal into a second EDFA and a second OF. The phase of an optical signal cannot be measured directly from the optical power, but it has to interfere with some known reference, which in our case was a preceding symbol. Thus, we measured a differential phase of pulses in the spirit of differential phase-shift-keyed (DPSK) demodulated output using a delay-line interferometer (DLI, by Kylia) with free spectral range of 1 GHz. A single output of the DLI was measured with a p-i-n photodiode (HP 11982A) that was connected to a BERT (Advantest D3286). The expected bit sequence of the DPSK modulated output was preprogrammed into the BERT, which counted the errors.

In this setup the ratio between optical injection and optical output^21^ *P*_inj_/*P*_*out*_ ≈ 4 dB. We permitted the positive ratio, because our objective was to optimize the BER, not the power budget. In practice, BER was in minimum when the slave VCSEL was pumped only slightly above its lasing threshold that in turn enabled the maximal speed of operation. Indeed, the power budget, the speed of operation, and the achieved BER are interdependent; and furthermore, they are dependent on internal laser properties, such as the linewidth enhancement factor *α*, the decay rate of the cavity field, and the decay rate of the carrier number. In our experiment we optimized the BER in expense of the power. This should not be interpreted as a statement concerning the power budget of laser based normalization operations, merely just specifics of this particular experiment. By use of another objectives of the experiment, and possibly another type of a laser, one may tailor and optimize the performance. To optimize the power budget the linewidth enhancement factor *α* of a laser should be minimized–preferably to nil.

As known in the art, the optimal regime of injection locking, and thus normalization operations, is obtained when the detuning between ECL master and VCSEL slave approaches zero. We optimized the injection locking by setting the wavelength of the ECL close to free-running wavelength of the VCSEL and then detuning was minimized by adjusting the VCSEL pump current (as the amount of pumping is known to determine the emission wavelength of a laser diode). While the semiconductor laser locking and thus the emission properties are highly dependent on the amount of detuning between the master and slave lasers, the fine-tuning of the pump power enables minimization of the BER. To avoid thermal drift of the VCSEL free-running wavelength due to environmental conditions, temperature of the VCSEL wafer was controlled with the TEC system. In free-running state the VCSEL emission has a vastly broader bandwidth than the ECL, yet, as known in the art, the slave adopts, or at least imitates, the properties of the master^21^. As a consequence, in the locked state the VCSEL emission bandwidth resembles the one of the ECL. Depending on both the pump current and the resulting detuning, an optimal optical injection power was set to minimize the BER of the received demodulated DPSK signal.

The measurement of the BER was performed by gradual degradation of the signal quality by increase of the ER of the hybrid encoded symbols. To compare the injection locked results with the non-injection locked reference, we measured the BER with and without the presence of the slave laser. In practice, the measurement setup had a 50/50 coupler whose other arm was used as the reference (dashed arrow in Fig. 5) that at times was connected to the input of the DPSK receiver (open dot in Fig. 3) instead of the optical output of the circulator.

## Supplementary information

Supplementary Information
