# Supplementary material for: All-optical majority gate based on an injection-locked laser
Source: Sci Rep. 2019 Oct 10;9:14576. doi: 10.1038/s41598-019-51025-y (PMC6787056; doi:10.1038/s41598-019-51025-y)
Supplement: Supplementary file 1 — Supplementary Information [file 41598_2019_51025_MOESM1_ESM.pdf]

Supplementary Information

All-optical majority gate based on an injection-locked laser

Tuomo von Lerber[1,2,5,*], Matti Lassas[2], Vladimir S. Lyubopytov[1,3], Lauri Ylinen[2], Arkadi Chipouline[5,1], Klaus Hofmann[4], and Franko Küppers[5,1]

[1]Photonics Lab, Technische Universität Darmstadt, Germany
[2]Department of Mathematics and Statistics, University of Helsinki, Finland
[3]Telecommunication Systems Dept., Ufa State Aviation Technical University, Russian Federation
[4]Integrated Electronic Systems Lab, Technische Universität Darmstadt, Germany
[5]Center for Photonics and Quantum Materials, Skolkovo Institute of Science and Technology, Moscow, Russian Federation
[*]tuomo.lerber@iki.fi

ABSTRACT

This Supplementary Information contains two sections. In the first section we will show that in steady-state a weakly injection-locked laser can perform a two-dimensional complex normalization operation of linearly polarized electric fields. In the second section we study cascadability with simulation of an optical full adder circuit and a 3-bit ripple-carry adder circuit based on semiconductor laser rate equations.

S1 Steady-state analysis of an injection-locked semiconductor laser

In following we will show that in steady-state a weakly injection-locked laser can perform a two-dimensional complex normalization operation of linearly polarized electric fields. That is, the electric field of the emitted light adopts the master polarization and frequency, and locks the phase, while it regenerates the amplitude. This relation can be written as

$$\bar{E} = \bar{u}\,\|\bar{u}\|^{-1} e^{i\theta}\,\sqrt{\mu - 1}, \tag{1}$$

where μ is the pumping rate of the laser ($\mu = 1$ at the threshold), $\bar{E}, \bar{u} \in \mathbb{C}^2$ are the slowly varying electric field amplitudes of the slave and the master, respectively. The angle θ is the phase-shift between input and output signals. The carrier waves are taken to be of the form $\exp\left(-i\left(kz - \omega t\right)\right)$.

In absence of laser cavity anisotropies, the electric fields with slowly varying amplitude $E_{x,y}(t)$ can be modeled with a set of rate equations[44]

$$\begin{aligned}
\dot{E}_x &= -\gamma_c E_x - i\gamma_c \alpha E_x + \gamma_c (1 + i\alpha)(NE_x + inE_y) + \gamma_c \eta u_x, && (2)\\
\dot{E}_y &= -\gamma_c E_y - i\gamma_c \alpha E_y + \gamma_c (1 + i\alpha)(NE_y - inE_x) + \gamma_c \eta u_y, && (3)\\
\dot{N} &= -\gamma\left[N\left(1 + |E_x|^2 + |E_y|^2\right) - \mu + in\left(E_y E_x^* - E_x E_y^*\right)\right], && (4)\\
\dot{n} &= -\gamma_s n - \gamma\left[n\left(|E_x|^2 + |E_y|^2\right) + iN\left(E_y E_x^* - E_x E_y^*\right)\right], && (5)
\end{aligned}$$

where N is a difference of normalized upper and lower level populations, n is a normalized value of the difference between the population inversions, γ_c is the decay rate of the electric field in the cavity, i.e., $(2\gamma_c)^{-1}$ is the cavity photon lifetime; α is the linewidth enhancement factor, γ is the decay rate of the total carrier number, γ_s is a decay rate that is related to electron angular momentum, η is the coupling efficiency, and $u_{x,y}$ is the injected field. In following we shall assume that the laser operates close to threshold $1 < \mu < 1.2$, that the input $u_{x,y}$ and the laser electric field $E_{x,y}$ polarization components have the same carrier frequencies, and that the coupling is weak $\|\bar{u}\| \ll \|\bar{E}\|$.

At the steady-state equations (2-5) become zero and we can re-arrange the terms of equation (5) as

$$n = -i\gamma N \frac{E_y E_x^* - E_x E_y^*}{\gamma_s + \gamma \|E\|^2}. \tag{6}$$

For linearly polarized field the difference $E_y E_x^* - E_x E_y^* = 0$ and thus $n = 0$ for steady-state. The remaining steady-state equations can be written as

$$E_{x,y} = u_{x,y}\eta(1+i\alpha)^{-1}(1-N)^{-1}, \quad (7)$$
$$N = \mu\left(1+\|E\|^2\right)^{-1}. \quad (8)$$

To solve N of equation (8) we calculate $\|E\|^2$ from equation (7) and get

$$N + N\|\bar{u}\|^2\eta^2(1+\alpha^2)^{-1}(1-N)^{-2} - \mu = 0. \quad (9)$$

The cubic polynomial of $(N-1)$ has a pair of roots in immediate vicinity of zero and we can approximate the equation with a second degree Taylor polynomial in the neighbourhood of $N-1=0$. We have an approximation

$$N = 1 - \frac{\eta\|\bar{u}\|}{\sqrt{1+\alpha^2}\sqrt{\mu-1}}, \quad (10)$$

and when substituted into equation (7) we get

$$E_x = u_x\|\bar{u}\|^{-1}\sqrt{\mu-1}\exp\left[-i\mathrm{Arg}\left(1+i\alpha\right)\right], \quad (11)$$
$$E_y = u_y\|\bar{u}\|^{-1}\sqrt{\mu-1}\exp\left[-i\mathrm{Arg}\left(1+i\alpha\right)\right],$$

where we recall that $E = (E_x, E_y)$, $u = (u_x, u_y)$, and $\|u\| = (|u_x|^2 + |u_y|^2)^{1/2}$.

For the purpose of this work the absolute phase $\theta = -\mathrm{Arg}\left(1+i\alpha\right)$ is of little interest, given that it stays constant in respect to the injected field. The amplitude multiplying coefficient $\sqrt{\mu-1}$ is important.

Summarising the above, we write the above relation in vectorial form as

$$\bar{E} = \bar{u}\|\bar{u}\|^{-1}e^{i\theta}\sqrt{\mu-1}, \quad (12)$$

which is an amplified normalization of complex vector $\bar{u} \in \mathbb{C}^2$. In other words, the injection-locked semiconductor laser adopts the linearly polarized master electric field and regenerates its amplitude. When the optical power of the injection approaches the optical power of the free-running laser, the laser output power will change, yet, the fluctuation in the input will typically be strongly quenched.

S2 Optical full adder and ripple-carry adder

We studied cascadability by simulation of an optical full adder consisting of three majority gates; that is, three semiconductor lasers. The circuit was modelled after a known digital counterpart illustrated schematically in Fig. S1a. A schematic optical implementation of the full adder is depicted in Fig. S1b. Component-wise the optical circuit consists of three lasers, a number of directional couplers, and phase controls, yet, for the sake of clarity only the phase reversing phase controllers are shown. The purpose of directional couplers following the lasers M2 and M3 is just to attenuate electric field amplitudes by a factor of $1/\sqrt{2}$ to ensure balanced optical powers at all lasers and to ensure equal power at the circuit input and output ports. In similar fashion we simulated also an optical ripple-carry adder of three bits (see Fig. S1c) using the full adders. This simulation consisted of nine lasers.

Electric field amplitudes of the Boolean inputs A, B, and C_{in} are denoted as aE, bE, and $c_{in}E$, respectively, where $a, b, c_{in} \in \{-1, +1\}$ and $E = [e_x, 0]^T$ for some $e_x \in \mathbb{C}\setminus\{0\}$. The lasers were modelled using differential rate equations[44] with following parameters: Linewidth enhancement factor $\alpha = 3$, decay rate of the cavity electric field $\gamma_c = 10^{12}$ s^{-1}, decay rate of the carrier number $\gamma = 10^9$ s^{-1}, excess in the spin decay rate $\gamma_s = 50\cdot 10^9$ s^{-1}, linear phase anisotropy $\gamma_p = 0$ s^{-1}, and amplitude anisotropy $\gamma_a = 0$ s^{-1}. At steady-state the emitted electric field amplitude of each laser was set to $\sqrt{2}E$ where the injection current satisfies $\mu - 1 = 2\|E\|^2$. In these simulations $\mu = 1.2$. In this modelling, the optical media is assumed lossless and all losses are accumulated into laser power coupling efficiency $\eta_P = -14$ dB, which aggregates losses from the couplings and from the optical media. In addition, every directional coupler is assumed to divide (and combine) an electric field amplitude in two arms with multiplication factor of $1/\sqrt{2}$.

Simulated operation of the full adder is shown in Fig. S2. The simulation was performed over time period of $t = [-2, 9]$, yet, the plot omits the first nanosecond. The electric field amplitudes of inputs A, B, and C_{in} were zero during the periods $t = [-2, 0)$ and $t = [8, 9]$ (marked with grey in the figure). Consequently, the laser outputs are chaotic. During the time period of $t = [0, 8)$, the triple $(\mathrm{A}, \mathrm{B}, \mathrm{C_{in}})$ of Boolean inputs cycles through the set $\{0, 1\}^3$ of possible values, staying constant for 1 ns at a time. This is shown in the three topmost graphs in Fig. S2.

The Boolean inputs A, B and C_{in}, and the outputs S and C_{out} of a full adder are related by $A+B+C_{in} = C_{out}S$, where the right-hand side is interpreted as binary representation of the integer $C_{out} \cdot 2 + S$. In Fig. S2, the fourth and the fifth graphs represent the phase of the output fields S and C_{out}. The phase values corresponding to Boolean values of 0 and 1 are $-\pi/2$ and $\pi/2$, respectively. After a change in an input, the amplitudes and phases of the outputs will stabilize to correct steady-state values.

In Fig. S3, simulated operation of a three-bit ripple-carry adder is shown. A ripple-carry adder with inputs A_i and B_i, and outputs S_i and C_{out} ($i = 1, 2, 3$) performs a calculation $A_2A_1A_0 + B_2B_1B_0 = C_{out}S_2S_1S_0$, where the summands and the right-hand side are binary representations of integers. The ripple-carry adder is composed of three full adders in cascade, where the first and second full adders have their C_{out} outputs connected to the C_{in} inputs of the second and third full adders, respectively (see Fig. S1c). The C_{in} of the first full adder is set to 0, and the C_{out} of the third full adder is the C_{out} of the whole ripple-carry adder.

In this simulation, the time period was also $t = [-2, 9]$, and the first nanosecond is omitted from the plot. The amplitudes of the input fields were zeros during the time periods of $[-2, 0)$ and $[8, 9]$. During the active time period $[0, 8)$, the inputs A_0, A_1 and A_2 were 1, and the inputs B_1 and B_2 were 0. For the first half of the active time period the input B_0 was 0, and for the second half it was 1. The value of B_0 as a function of time is depicted in the first graph in Fig. S3.

On the time interval $[0, 4)$ all three full adders compute $1+0+0 = 01$. After the change of the input B_0 at time $t = 4$, the first full adder computes $1+1+0 = 10$, which causes its C_{out} bit to flip from 0 to 1. Therefore the second full adder computes $1+0+1 = 10$, and hence also its C_{out} bit flips from 0 to 1. This in turn causes the third full adder to flip its C_{out} bit as well. In this way the carry-bit "ripples" through the circuit all the way to the C_{out} output of the circuit.

Graphs of phases of S_i and C_{out} in Fig. S3 show that after the change in input B_0, all output fields stabilise to the expected value. It takes longer for the output fields of S_1, S_2 and C_{out} to stabilise compared to S_0. This is due to the fact that each full adder in the cascade is influenced by the previous full adders, but not vice versa. For example, the electric field of the output S_2 is influenced by a total of five lasers from the circuit; lasers M1 from the first two full adders, and all three lasers from the last full adder.

References

44. Martin-Regalado, J., Prati, F., San Miguel, M. & Abraham, N. Polarization properties of vertical-cavity surface-emitting lasers. *IEEE J. Quantum Electron.* **33**, 765–783 (1997).

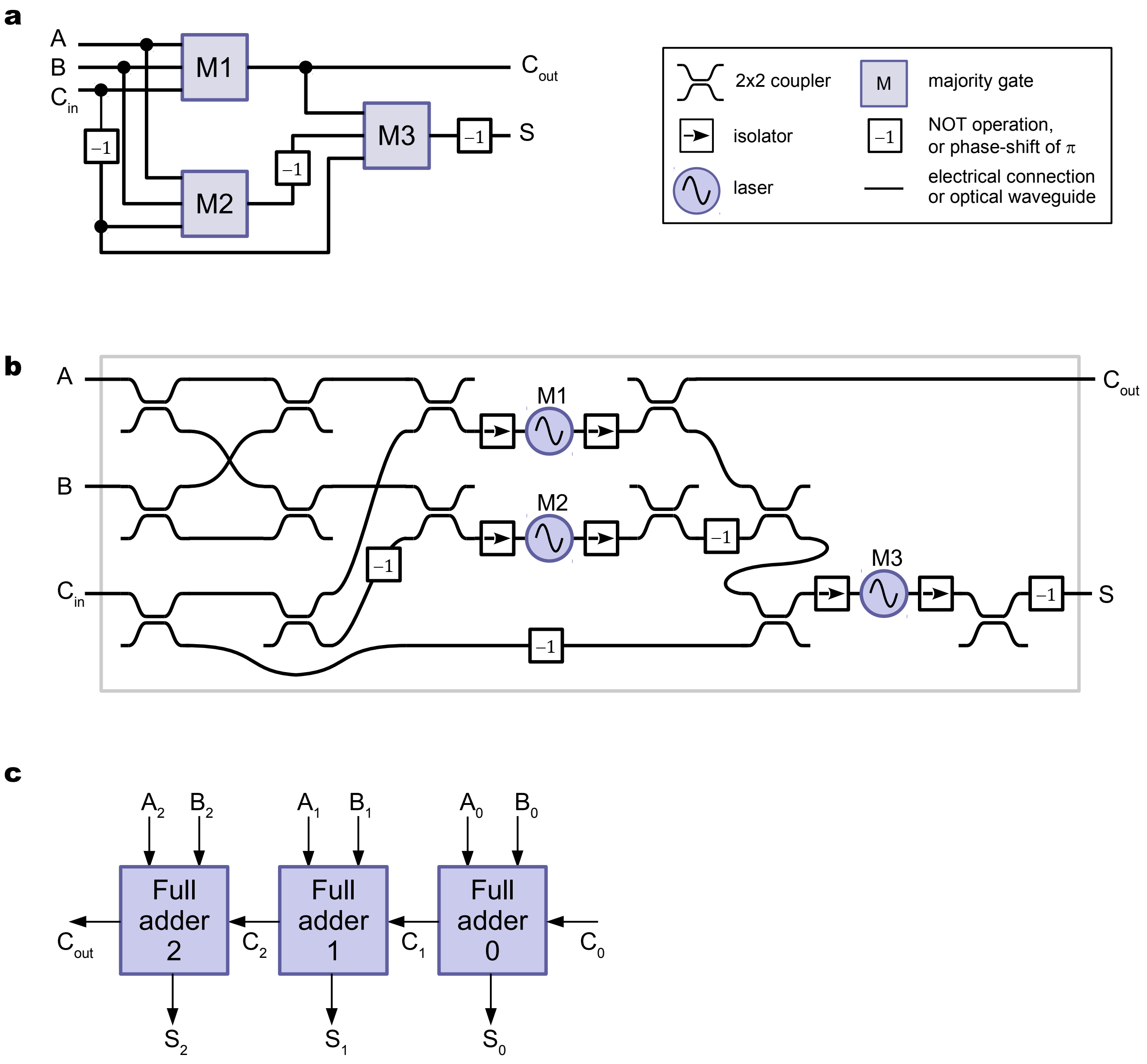

Figure S1. Full adder circuits based on three majority logic gates. a, Digital electronics full adder. b, Schematic illustration of an optical equivalent full adder circuit based on three lasers. Operational stability of the lasers and the unidirectional flow of information is ensured with pairs of isolators that surround the lasers. Alternatively, one could use optical circulators, or instead of using Faraday effect devices, employ unidirectional ring-lasers. c, Simulated ripple-carry adder of nine lasers.

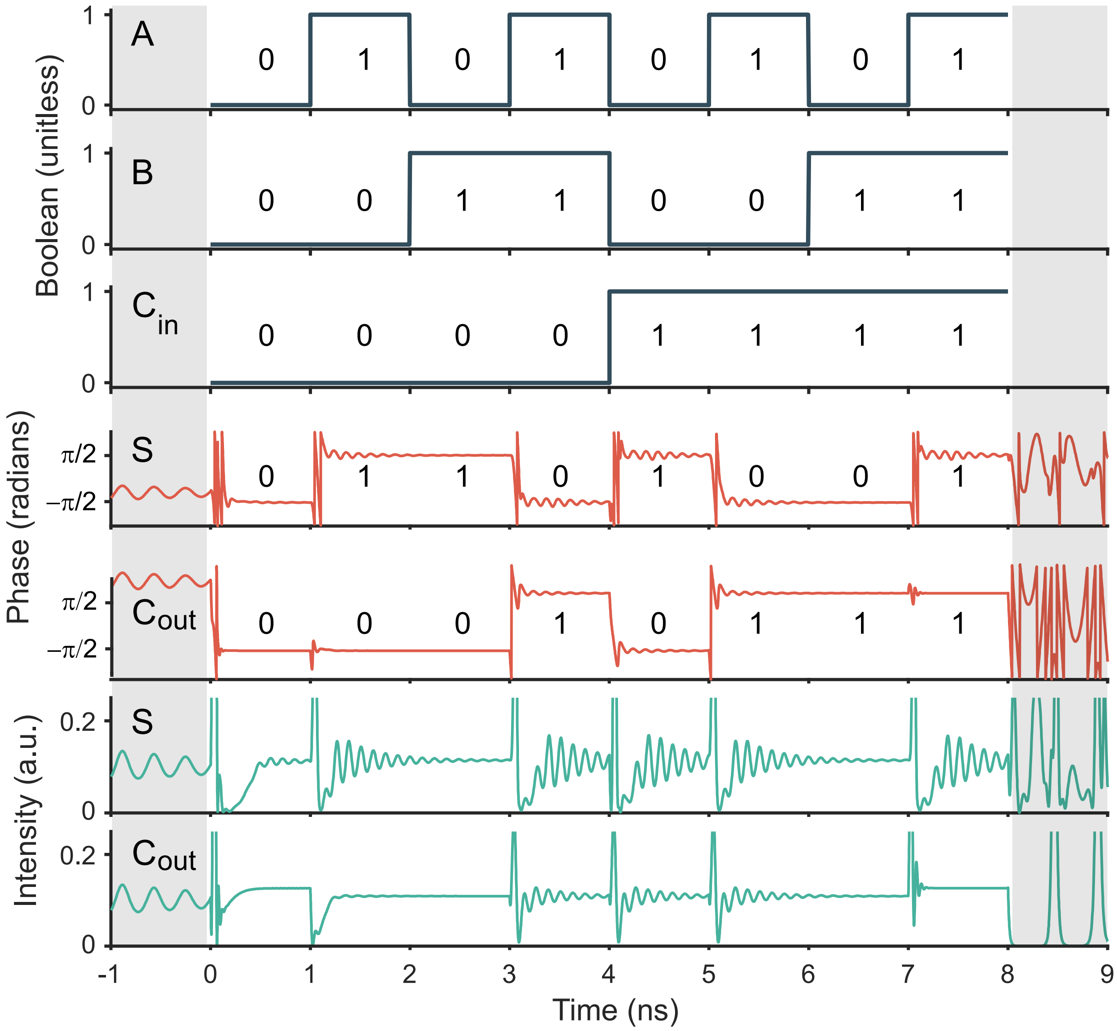

Figure S2. Simulated operation of a full adder. For the purpose of illustrations the Boolean symbol values of 0 and 1 are shown by electric field phases of $-\pi/2$ and $\pi/2$, respectively.

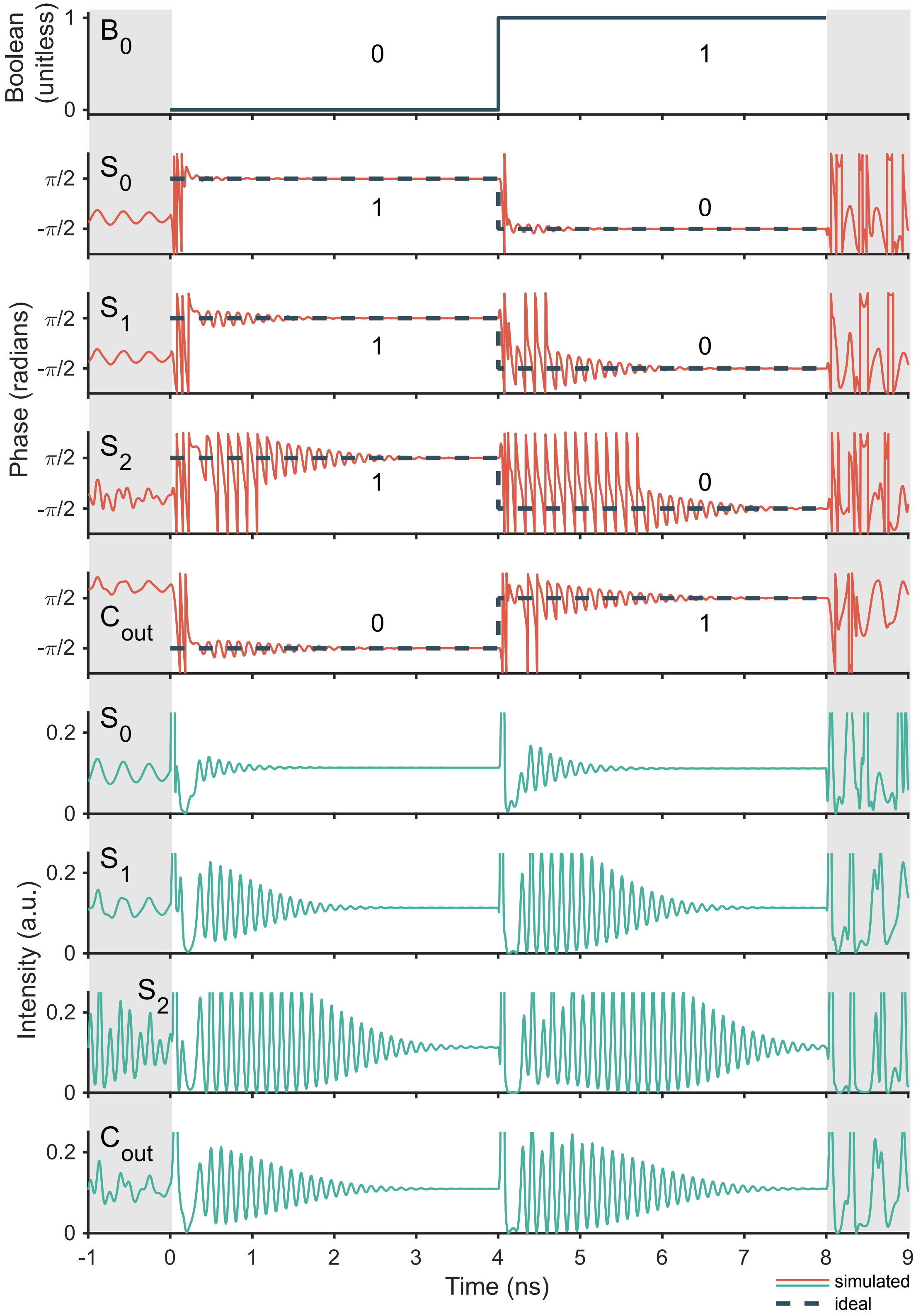

Figure S3. Simulated operation of an optical ripple-carry adder. For the purpose of illustrations the Boolean symbol values of 0 and 1 are shown by electric field phases of $-\pi/2$ and $\pi/2$, respectively.
